# Insulin-like growth factor 2 mRNA-binding protein 2-stabilized long non-coding RNA Taurine up-regulated gene 1 (TUG1) promotes cisplatin-resistance of colorectal cancer via modulating autophagy

**DOI:** 10.1080/21655979.2021.2012918

**Published:** 2022-01-11

**Authors:** Cuifeng Xia, Qiang Li, Xianshuo Cheng, Tao Wu, Pin Gao, Yongfang Gu

**Affiliations:** a Department of Colorectal Surgery, The Third Affiliated Hospital of Kunming Medical University (Yunnan Cancer Hospital), Kunming, Yunnan, China; b Department of Hepatobiliary Surgery, The Second People’s Hospital of Qujing, Qujing, Yunnan, China

**Keywords:** IGF2BP2, lncRNA TUG1, colorectal cancer, HDGF, β-catenin

## Abstract

Long non-coding RNAs (lncRNAs) have been demonstrated to influence the chemoresistance of colorectal cancer (CRC). Therefore, the study is designed to investigate the regulatory function and mechanism of Taurine up-regulated gene 1 (TUG1) in the cisplatin resistance of CRC. qRT-PCR checked the expressions of TUG1, Insulin-like growth factor 2 mRNA-binding protein 2 (IGF2BP2), and miR-195-5p in CRC tissues and cells. The TUG1 or miR-195-5p overexpression model was engineered in CRC cells, followed by treatment with DDP or the autophagy inhibitor (Chloroquine, CQ). CCK8 (Cell Counting Kit-8) and the colony formation experiment monitored cell proliferation. Flow cytometry examined apoptosis, Transwell tracked migration and invasion, and Western blot ascertained the protein profiles of autophagy proteins (LC3I/LC3II and Beclin1) and the HDGF/DDX5/β-catenin pathway. Dual-luciferase gene reporter assay and RNA immunoprecipitation confirmed the binding correlation between TUG1 and miR-195-5p and between miR-195-5p and HDGF. Furthermore, *in-vivo* experiments in nude mice probed the function and mechanism of IGF2BP2 in CRC cell growth. The profiles of TUG1 and IGF2BP2 were elevated in CRC tissues, and IGF2BP2 enhanced TUG1’s expression in CRC cells. TUG1 activated autophagy to facilitate CRC cells’ resistance to DDP. TUG1 targets miR-195-5p, and miR-195-5p targets HDGF. Overexpression of miR-195-5p abated the cancer-promoting function of TUG1 and curbed the profile of the HDGF/DDX5/β-catenin axis. TUG1 stabilized by IGF2BP2 boosted CRC cell proliferation, migration, migration, and autophagy via the miR-195-5p/HDGF/DDX5/β-catenin axis, hence enhancing CRC cell’s resistance to DDP.

## 1. Introduction

Colorectal cancer is a prevailing malignancy in the digestive system [1]. Colorectal cancer (CRC) is a disease inextricably associated with heredity, environment, and living habits [2,3]. Reportedly, the incidence rate of CRC presents a younger trend, and patients in the advanced stage manifest a poor prognosis [4,5]. Cisplatin (DDP) treatment earns a significant place among all the therapies for colorectal cancer, but the increasing resistance of cisplatin has become a commonplace cause of CRC recurrence [6]. The study is focused on the molecular mechanism of CRC cisplatin resistance so as to provide reliable grounds for inverting cisplatin resistance existing in CRC.

Long non-coding RNAs (lncRNAs), usually defined as RNA molecules that consist of 200 nucleotides, can serve as oncogenes or tumor suppressor genes to modulate the occurrence and progression of diverse tumors [7]. The functions of lncRNAs are closely correlated with their subcellular locations: lncRNAs can modulate not only the epigenetic and transcriptional levels of genes in the nucleus but also the genes’ post-transcriptional and translational levels in the cytoplasm [8]. Thus, lncRNA has a potent role in controlling the progression of tumors [9,10]. Additionally, lncRNAs also participate in the regulation of multiple tumors’ resistance or sensitivity to chemotherapy drugs [11]. For instance, lncRNA metastasis-associated lung adenocarcinoma transcript 1 (LncRNA MALAT1) acts as a sponge of miR-200a to enhance the proliferation of lung cancer cells and their resistance to gefitinib [12]. LncRNA taurine up-regulated 1 (TUG1) is critical to tumor drug resistance [13]. TUG1 boosts the resistance of ESCC cells to DDP via Nrf2 up-regulation [14]. However, we are still in the dark about the function and mechanism of TUG1 in CRC cisplatin resistance.

microRNAs (miRNAs), a type of small non-coding RNAs with conserved evolution, can modulate the translation or degradation of the target mRNA to influence disease progression [15]. lncRNAs can modulate the profiles of miRNAs acting as a sponge/bait, hence controlling the regulatory functions of miRNAs and then affecting tumor progression [16,17]. Overexpression of lncRNA Deleted in Lymphocytic Leukemia 2 (lncRNA DLEU2) impairs miR-30 c-5p’s restraint on the profile of SOX9 to facilitate non-small cell lung cancer cell growth both *ex vivo* and *in vivo*, bringing into full play its oncogenic effects [18]. It’s worth noting that BRAF-activated non-protein coding RNA (lncRNA BANCR) initiated by BRAF triggers the Wnt/β-catenin pathway via the profile of the sponge miR-195-5p so as to bolster the proliferation, migration, and invasion of pancreatic cancer cells [19]. miR-195 curbs Coactivator-associated arginine methyltransferase 1 (CARM1) to strengthen the radiosensitivity of CRC cells [20]. Given these findings, we have confidently surmised that lncRNA TUG1 modulates the profile of the target miR-195 to influence CRC cells’ resistance to cisplatin.

Hepatoma-derived growth factor (HDGF) is a heparin-binding protein that has been discovered to present an aberrant expression in multiple cancers and partake in the modulation of malignant cancer cell behaviors like apoptosis, metastasis, and angiogenesis [21]. Overexpression of HDGF can suppress nordihydroguaiaretic acid (NDGA)-elicited CRC cell apoptosis and tumor growth, hence boosting CRC resistance to NDGA [22]. DEAD-box RNA helicase DDX5 (DDX5) can participate in transcription factor activation to function in cancer occurrence and progression [23]. Pumilio RNA-binding family member 1 (PUM1) interacts with DDX5 and positively modulates its expression, and their knockdown can repress cetuximab-resistant colon cancer cells’ sensitivity to cetuximab [24]. Therefore, targeting HDGF and DDX5 is a promising treatment against CRC.

By regulating lncRNAs, RNA-binding proteins (RBPs) can exert biological functions [25]. The insulin-like growth factor-2 mRNA-binding protein (IGF2BP) family members, IGF2BP1-3 for one, all play essential functions in embryogenesis, carcinogenesis, and chemoresistance by affecting non-coding RNAs’ stability, translatability, or localization [26–28]. For instance, IGF2BP2 interacts with and positively regulates DANCR by functioning as a reader for m6A modified DANCR and stabilizing DANCR RNA [29]. Here, we detected IGF2BP2 and lncRNA-TUG1 in cisplatin-resistant CRC cells and discovered that both IGF2BP2 and TUG1 were substantially up-regulated. Down-regulation of lncRNA-TUG1 contributed to reduced cisplatin resistance and enhanced miR-195-5p expression. Moreover, IGF2BP2 down-regulation attenuated TUG1’s expression. Therefore, we conjectured that IGF2BP2-mediated lncRNA-TUG1 served as a sponge of miR-195-5p to boost the growth of CRC cells and enhance their resistance to cisplatin. We hope this study provides a new reference in treating CRC.

## 2. Materials and Methods

### 2.1 Collection and treatment of clinical specimens

From February 2018 to May 2019, tumor tissue samples and adjacent normal tissue samples (43 cases each) were collected from the patients who were first diagnosed with colorectal cancer in our hospital [30]. The specimens were preserved in the RNA storage solution for the following experiments. None of the patients had received chemotherapy or radiotherapy preceding surgery or had any other malignancies or severe diseases. Furthermore, all of them had signed the informed consent for the study of their own accord.

### 2.2 Cell culture

Human colorectal cancer cell lines (LoVo, LS513, HT-29, HCT15, DLD-1) and human normal colorectal mucosal cells (FHC), ordered from the Cell Bank of Chinese Academy of Sciences (Shanghai), were seeded onto a Dulbecco’s minimal essential medium/Ham’s-F12 (Thermo Fisher Scientific, Shanghai, China) medium incorporating 10% fetal bovine serum (Hyclone, Logan, UT, USA) and 100 U/mL penicillin (Sigma, St.Louis, Mo, USA). Then, the medium was incubated in an incubator at 37°C with 5% CO_2_ [31]. As the cells achieved 70 ~ 80% confluence, digestion, and passage were conducted employing 0.25% trypsin.

### 2.3 Cell transfection and treatment

LoVo and LS513 cells were gleaned in the logarithmic growth stage. GenePharma (Shanghai, China) took on the design and synthesis of the plasmid pcDNA-TUG1 and its negative control (vector), siRNA against TUG1 (si-TUG1) and the control (si-NC), plasmid pcDNA-IGF2BP2 and its negative control (vector), siRNAs against IGF2BP2 (si-IGF2BP2) and the control group (si-NC), as well as miR-195-5p mimics and the negative control group (miR-NC), which were used in the experiment. Lipofectamine®2000 (Invitrogen) was adopted to transfect these plasmids, mimics, and their respective negative controls into LoVo and LS513 cells [31]. Forty-eight hours later, qRT-PCR determined the efficiency of the transfection. For autophagy inhibition, the cells were dealt with CQ (20 μM), the autophagy inhibitor.

### 2.4 Quantitative real-time polymerase chain reaction (qRT-PCR)

Total RNA was extracted out of cells and tissues with the use of TRIzol reagent. The Revertra ACE qPCR RT kit (Toyobo, Osaka, Japan) was utilized for reverse transcription in accordance with the instructions. The miRNA was reversely transcribed adopting the hyperscript III miRNA first strand cDNA synthesis Kit (NovaBio, Shanghai, China). The SYBR Premixex Taq II kit (Beijing Zhijie Fangyuan Technology Co., Ltd.) was employed for amplification via the real-time fluorescence quantitative PCR analysis system. The relative profile of the target gene was calculated as per the formula 2^−∆∆CT^ [32]. U6 was taken as the internal reference of miR-195-5p, while GAPDH was taken as that of IGF2BP2 and TUG1. The primer sequences of the aforementioned target genes are detailed in Table 1.

**Table 1.** Primer sequences in RT-PCR

Genes	Primer sequences	
**miR-195-5p**	F: GAATTCGCCTCAAGAGAACAAAGTGGAG	R: AGATCTCCCATGGGGGCTCAGCCCCT
**U6**	F:GCTTCGGCAGCACATATACTAAAAT	R:CGCTTCACGAATTTGCGTGTCAT
**IGF2BP2**	F: TCTGTCTGGCCTGAGAAGTG	R: AACACAGACACAGAAACCGC
**TUG1**	F:CTGGAGTGGAGGGCTGTTAA	R: GTACCTCCACTCAGCACAGT
**GAPDH**	F: TGGTTGAGCACAGGGTACTT	R: CCAAGGAGTAAGACCCCTGG

### 2.5 Cell Counting Kit-8 (CCK8)

LoVo and LS513 cells were inoculated into 96-well plates with 3000 cells per well. The plates were put in an incubator at 37°C and with 5% carbon dioxide (CO_2_). On the 24^th^, 48^th^, and 72^nd^ hour of cell culture, a microplate reader was exploited to check the optical density (OD) value of each well and calculate the cell viability of each group after CCK-8 reagent (MedChem Express, New Jersey, USA) was adopted for two hours’ incubation [33].

### 2.6 Colony formation assay

LoVo and LS513 cells were seeded into Petri dishes (60 mm) with 800 cells for each and then grown in an incubator under the conditions of 37°C and 5% CO_2_. Two weeks on, the cells were dyed employing the crystal violet solution (0.1% crystal violet) and counted subsequent to observation [32].

### 2.7 Transwell assay

The bottoms of the Transwell chambers were coated with Matrigel (356,234, Beijing Qiyan Biotechnology Co., LTD) at a concentration of 1:8. The suspension of LoVo and LS513 cells resuspended in a serum-free medium (over 100 ul) was scattered evenly in the upper Transwell compartment, with a 500 μl DMEM high-glucose medium administered to the lower chamber for 48 hours’ culture. After the medium in the upper room was removed, 4% paraformaldehyde was employed to immobilize the cells for 30 minutes, which were then stained with 0.1% crystal violet reagent for an hour. A microscope was harnessed to observe and count the stained cells for cell invasion assessment [31]. The cell migration capability assay: Matrigel was not used for coating beforehand, but the other procedures were the same as those in the cell invasion assay.

### 2.8 Western blot

Total protein was extracted out of the cells and tissues. The BCA protein concentration kit was adopted to quantify the proteins, and the bromophenol indicator was taken to prepare the sodium dodecyl sulfate-polyacrylamide gel electrophoresis (SDS-PAGE) samples. With 5% concentrate gel and 10% separation gel, 20 μL of the protein samples were loaded. Following electrophoresis, the proteins were moved onto polyvinylidene difluoride (PVDF) membranes (Millipore, Bedford, MA, USA) at a constant current of 200 mA. After being sealed with 10% skimmed milk powder solution for 2 hours [33], the membranes were incubated along with primary antibodies Anti-IGF2BP2 (1:2000, ab129071), Anti-LC3 (1:2000, ab192890), Anti-Beclin1 (1:2000, ab207612), Anti-HDGF (1:2000, ab128921), Anti-DDX5 (1:10,000, ab126730), Anti-β-catenin (1:1000, ab68183), Anti-β-actin (1:2000, ab8227), Anti-p53 (1:2000, ab26), Anti-Bax (1:1000, ab32503), Anti-Bcl2 (1:2000, ab32124), and Anti-cleaved Caspase3 (1:1500, ab32351) overnight at 4°C. TBS with Tween-20 (TBST) was utilized to flush the membranes three times, 5 minutes each, which were then incubated together with the secondary antibody Goat Anti-Rabbit IgG (1:2000, ab6721) for two hours at indoor temperature. The membranes were rinsed in TBST another three times. The ECL chemiluminescence solution was applied for the color development of the protein samples. All the antibodies here were acquired from Abcam (MA, USA).

### 2.9 Dual luciferase reporter gene assay

Promega was responsible for the synthesis of the luciferase reporter vectors: wild-type TUG1 (TUG1-WT), Mutant-TUG1 (TUG1-MT), HDGF-WT, and HDGF-MT. LoVo and LS513 cells in the logarithmic growth stage were seeded into 96-well plates with a density of 3 × 10^4^ cells/well and cultured for 24 hours in an incubator. Then, Lipofectamine®2000 (Invitrogen) was exploited to transfect the above vectors together with miR-NC or miR-195-5p mimics into LoVo and LS513 cells. Forty-eight hours subsequent to the transfection, the luciferase activity was gauged in line with the instructions of the manufacturer [31].

### 2.10 RNA immunoprecipitation (RIP) assay

The experiment was implemented with the assistance of the RIP™ RNA-Binding Protein Immunoprecipitation Kit (Merck Millipore). RIP lysis buffer was administered to lyse LoVo and LS513 cells, and RNA (miR-NC or miR-195-5p) magnetic beads were combined with the mouse anti-IgG antibody and the human anti-Ago2 antibody or human anti-IGF2BP2 antibody [34]. TUG1’s level was determined through qRT-PCR.

### 2.11 Tumor formation in nude mice

This experiment had received the green light from the Medical Ethics Committee of the Third Affiliated Hospital of Kunming Medical University. LoVo cells, steadily transfected along with the vector, IGF2BP2, IGF2BP2+ si-TUG1, and IGF2BP2+ miR-195-5p, were harvested for use. Under aseptic conditions, 0.9% normal saline was adopted to transform LoVo cells into the single-cell suspension, with the cell concentration adjusted to 5 × 10^7^/mL. Forty nude mice on a BALB/c background, 4 to 6 weeks of age, were ordered from the Animal Experimental Center of Kunming Medical University. They were randomized to four groups, each with ten mice. Pentobarbital (30 mg/kg) was employed to anesthetize the nude mice, and 0.2 ml LoVo cell suspension was subcutaneously transfused into their right axillary through a syringe. After the inoculation, we kept a close watch on the mentality, diet, activities, defecation, and other normal conditions of the animals. Following the inoculation, the long diameter (a) and short diameter (b) of their tumors were gauged every other week as per the formula (volume = 0.5× ab2). On the 28^th^ day, the mice were sacrificed employing high-concentration CO_2_, with their tumor tissues harvested and weighed [34]. The tumor tissues of five nude mice randomly chosen from each group were histopathologically examined.

### 2.12 Immunohistochemistry (IHC)

Tumor tissues were harvested and made into paraffin-embedded slices. As the slices were routinely dewaxed and hydrated, 0.01 mmol/L sodium citrate buffer solution was administered for 15 minutes’ high-pressure repair. After being cooled down naturally, they were flushed in phosphate-buffered saline (PBS) three times, three minutes for each. 3% H_2_O_2_ was dripped into the wet box for 10 minutes’ incubation, with the aim to eliminate the endogenous peroxidase activity. PBS was applied to wash the samples three times, three minutes for each. Then the slices were incubated overnight with the primary antibody KI67 (1:200, ab16667) at 4°C. Following three times of PBS washing (5 minutes each), the secondary antibody (1:1000, ab6721) was given for 30 minutes’ incubation at indoor temperature. PBS was utilized again for three times washing with 5 minutes for each, followed by DAB dyeing for three minutes and restaining with hematoxylin [34]. Then the slices were sealed. At last, a microscope was manipulated to observe and count the number of positive cells (brown) in three non-overlapping fields. The primary and secondary antibodies used in the experiment both came from Abcam (MA, USA).

### 2.13 TdT-mediated dUTP Nick-End Labeling (TUNEL) assay

Tumor tissues were made into paraffin slices. After being dewaxed and hydrated, the sections were incubated with the proteinase K solution for 20 minutes at 37°C. Next, they were incubated along with 50 μL TUNEL detection solution for 60 minutes at 37°C in darkness, with DAPI employed for nucleus staining later on [31]. The anti-fluorescence quenching solution was utilized for sealing. A fluorescent micro-mirror was taken to observe the apoptotic cells (tinted with fluorescent green) in three random and discontinuous fields.

### 2.14 Statistical analysis

GraphPad Prism 8 (GraphPad Software, USA) was taken for statistical analysis. The measurement data were exhibited as mean ± standard deviation (x ± s). An independent sample t-test was adopted to compare two different groups, while ANOVA was for the comparison among multiple factors. Pearson correlation analysis investigated the correlation between TUG1 and miR-195-5p, TUG1 and HDGF, TUG1 and DDX5, as well as miR-195-5p and HDGF. *P < 0.05* was regarded as statistically meaningful.

## 3. Results

### 3.1 lncRNA-TUG1 and IGF2BP2 were up-regulated in colorectal cancer

**Figure 1.**
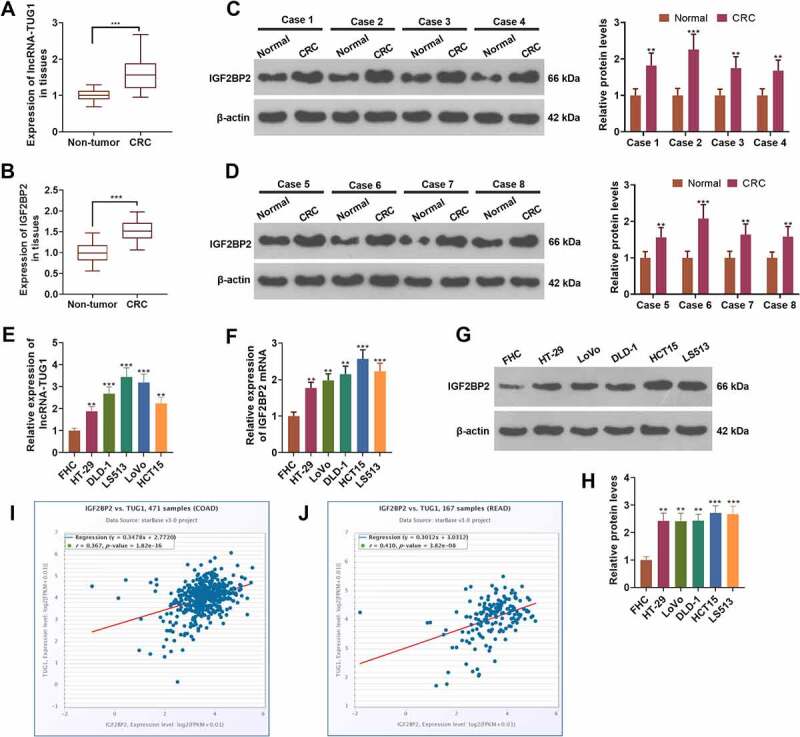
lncRNA-TUG1 and IGF2BP2 were up-regulated in colorectal cancer.

To evaluate the expression features of lncRNA-TUG1 and IGF2BP2 in colorectal cancer, we carried out qRT-PCR and Western blot. As shown by the outcomes, the profiles of lncRNA-TUG1 and IGF2BP2 were distinctly higher in cancer tissues as opposed to adjacent non-tumor tissues (*P < 0.05*, Figure 1(a-b)). Western blot displayed that IGF2BP2’s expression was remarkably higher in CRC tissues than in non-tumor tissues (*P < 0.05*, Figure 1(c-d)). qRT-PCR gauged lncRNA-TUG1 and IGF2BP2 mRNA expressions, revealing that their profiles were conspicuously higher in CRC cells (HT-29, DLD-1, LS513, LoVo, HCT15) than FHC cells (*P < 0.05*, Figure 1(e-f)). Western blot denoted that by contrast to FHC cells, CRC cells (HT-29, DLD-1, LS513, LoVo, HCT15) had enhanced IGF2BP2 protein level (*P < 0.05*, Figure 1(g-h)). The StarBase V3.0 database indicated that the profiles of lncRNA-TUG1 and IGF2BP2 were positively relevant both in colon adenocarcinoma (COAD) and rectum adenocarcinoma (READ) (Figure 1(i-j)). These findings signified that the profiles of lncRNA-TUG1 and IGF2BP2 were uplifted in colorectal cancer tissues and cells.

(a-b) qRT-PCR determined the relative profiles of lncRNA-TUG1 and IGF2BP2 in 43 cancer tissues and adjacent tissues. ***P < 0.001 (vs. the Normal group). (c-d) Western blot confirmed the protein profile of IGF2BP2 in eight pairs of CRC and normal tissues. (e-f) qRT-PCR checked lncRNA-TUG1 and IGF2BP2 mRNA expressions in CRC cells (HT-29, DLD-1, LS513, LoVo, HCT15) and FHC cells. (g-h) Western blot figured out the profile of IGF2BP2 in CRC and FHC cells. **P < 0.01, ***P < 0.001 (vs. the FHC group). N = 3. i-j: The correlation between lncRNA-TUG1 and IGF2BP2 in Colon adenocarcinoma (COAD) and rectum adenocarcinoma (READ) tissues was consulted through the ENCORI database (http://starbase.sysu.edu.cn/).

### 3.2 TUG1 strengthened colorectal cancer cells’ resistance to cisplatin via autophagy activation

**Figure 2.**
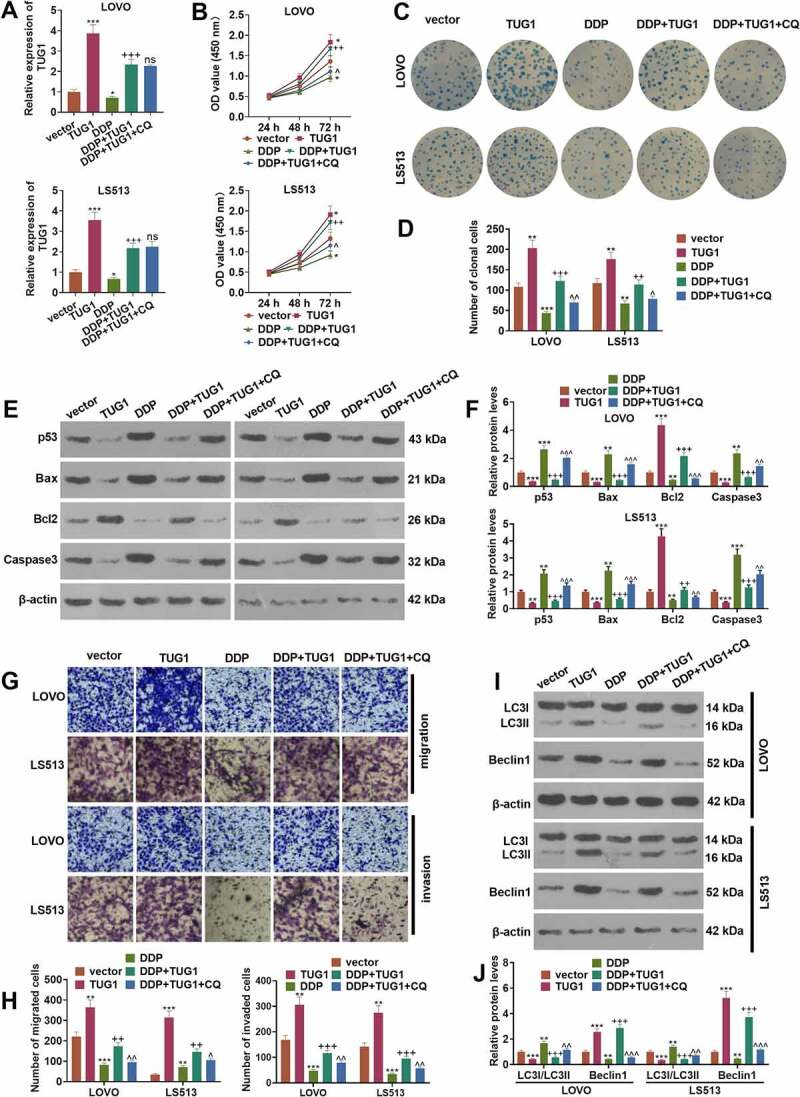
TUG1 triggered autophagy to boost cisplatin resistance in colorectal cancer cells.

To investigate whether TUG1 affects colorectal cancer cells’ proliferation and resistance to cisplatin *in vitro*, we utilized the vector and TUG1 to transfect CRC cells (LoVo and HCT15), respectively. Then, the autophagy inhibitor (Chloroquine, CQ) was administered to treat the cells, and the chemotherapy drug cisplatin (DDP) was taken for intervention. qRT-PCR revealed that overexpression of TUG1 inverted the inhibitory impact of DDP on TUG1 (*P < 0.05*, Figure 2(a)). In contrast with the DDP+TUG1 group, the profile of TUG1 had no significant alteration after CQ treatment (*P > 0.05*, Figure 2(a)). The proliferation and colony formation capabilities of CRC cells were assessed through CCK8 and colony formation assay, respectively. The data indicated that overexpression of TUG1 markedly enhanced the OD value and cell clone number of the cells, while DDP brought about the opposite situation. In contrast with the DDP group, TUG1 overexpression enhanced cell proliferation and cell colonies. In comparison with the DDP+TUG1 group, the OD value and the clone cell number were prominently lessened by CQ (*P < 0.05*, Figure 2(b-d)). Western blot examined apoptosis and unveiled that in contrast with the vector group or DDP group, p53, Bax, and cleaved Caspase3 were attenuated, and Bcl2 was bolstered followed by TUG1 overexpression. However, CQ exerted a pro-apoptotic function (vs. the DDP+TUG1 group, Figure 2(e-f)). As shown in Transwell assay, TUG1 overexpression vigorously facilitated CRC cell migration and invasion (vs. the vector or DDP group). When compared to the DDP+TUG1 group, CQ led to a substantial decline in the cells’ migration and invasion (*P < 0.05*, Figure 2(g-h)). Western blot confirmed the profiles of autophagy-associated proteins (LC3I/LC3II, Beclin1) in CRC cells. In contrast with the vector or DDP group, TUG1 overexpression contributed to a distinct reduction in the level of LC3I/LC3II and a rise in the profile of Beclin1. As opposed to the DDP+TUG1 group, CQ prominently elevated the profile of LC3I/LC3II and suppressed that of Beclin1 (*P < 0.05*, Figure 2(i-j)). These discoveries indicated that overexpression of TUG1 strengthened CRC cells’ survival and DDP resistance by repressing autophagy.

CRC cells (LoVo and HCT15) were transfected along with the vector and TUG1 overexpression plasmid, respectively. The cells were dealt with the autophagy inhibitor (Chloroquine, CQ) (20 µM) or Chemotherapy drug cisplatin (DDP) (4 µg/ml) for 24 hours. (a) qRT-PCR checked TUG1’s expression. (b) Cell proliferation was examined through CCK8 assay. (c-d) Colony formation assay assessed the colony formation ability of CRC cells. (e-f) Western blot verified the profiles of p53, Bax, Bcl2, and Caspase3 in LoVo and HCT15 cells. (g-h) Cell migration was examined via Transwell. (i-j) Transwell tracked cell invasion. (i-j) Western blot ascertained the profiles of autophagy-concerned proteins (LC3I/LC3II, Beclin1). *P < 0.05, **P < 0.01, ***P < 0.001 (vs. the vector group). *+P < 0.05, ++P < 0.01, +++P < 0.001* (vs. the DDP group). *^P < 0.05, ^^P < 0.01, ^^^P < 0.001* (vs.the DDP+TUG1 group). N = 3.

### 3.3 IGF2BP2 enhances the profile of lncRNA-TUG1

**Figure 3.**
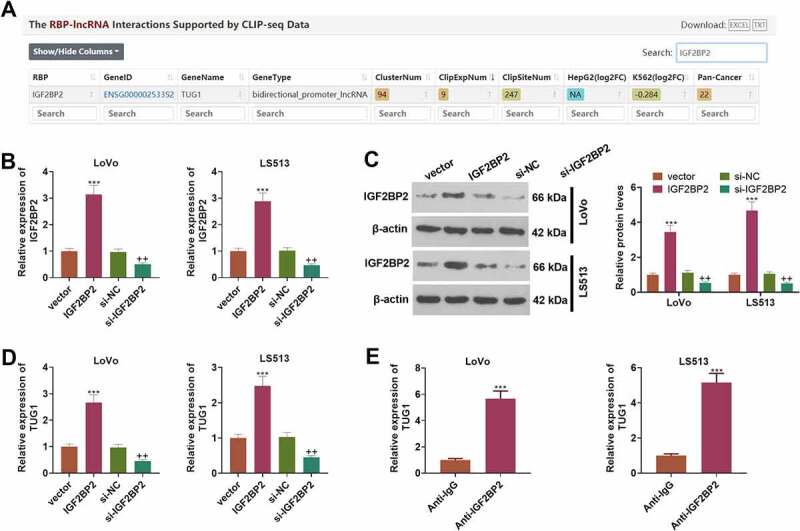
IGF2BP2 enhanced the profile of lncRNA-TUG1.

Through Starbase (also called ENCORI, https://starbase.sysu.edu.cn/), an online database, we discovered that IGF2BP2 contained a potential binding site with TUG1 (Figure 3(a)). To define the influence of IGF2BP2 on TUG1, we engineered the overexpression and knockdown models of IGF2BP2 in CRC cells (LoVo and HCT15) (*P < 0.05*, Figure 3(b)). Western blot unraveled that following the transfection of the pcDNA3.1-IGF2BP2 plasmids, the protein profile of IGF2BP2 was substantially lowered. On the contrary, the transfection of the plasmid si-IGF2BP2 gave way to a notable decline in its protein expression (*P < 0.05*, Figure 3(c)). As exhibited by qRT-PCR, overexpression of IGF2BP2 dramatically drove up the profile of TUG1, while IGF2BP2 knockdown vigorously curbed it (*P < 0.05*, Figure 3(d)). RIP validated the binding correlation between IGF2BP2 and TUG1, suggesting that TUG1 was markedly enriched in the anti-IGF2BP2 group as compared with the anti-IgG group (Figure 3E). These findings revealed that IGF2BP2 could boost TUG1’s expression in CRC cells.

(a) Starbase (also called ENCORI, https://starbase.sysu.edu.cn/) displayed that IGF2BP2 incorporated an underlying binding site with TUG1. (b) The overexpression and knockdown models of IGF2BP2 were engineered in CRC cells (LoVo and HCT15), respectively. (c) Western blot determined the protein profile of IGF2BP2. (d) TUG1’s expression was ascertained through qRT-PCR. (e) RIP assay validated the binding correlation between IGF2BP2 and TUG1. **P < 0.01, ***P < 0.001 (vs. the vector group). *++P < 0.01* (vs. the si-NC group). ***P < 0.001 (vs. the anti-IgG group). N = 3.

### 3.4 IGF2BP2/TUG1 enhanced HDGF and DDX expression in colorectal cancer cells

**Figure 4.**
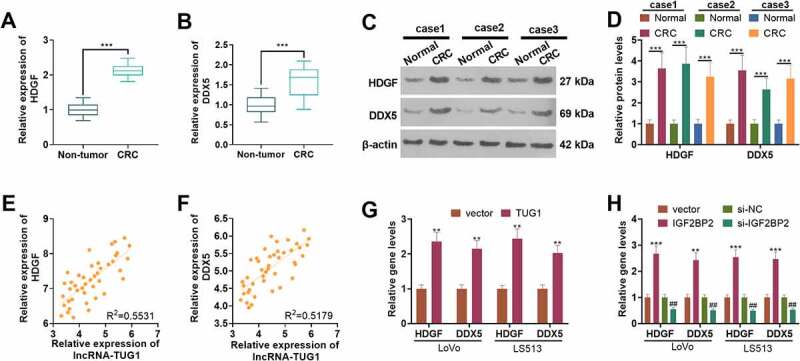
The expression features of HDGF and DDX in colorectal cancer.

qRT-PCR determined HDGF and DDX expressions in 43 pairs of tissues. HDGF and DDX expressions were considerably up-regulated in CRC tissues as compared with normal tissues (*P < 0.05*, Figure 4(a-b)). Western blot disclosed that their protein profiles were evidently higher than those in non-tumor tissues (*P < 0.05*, Figure 4(c-d)). As presented in Figure 4(e,f), the profiles of TUG1 and HDGF as well as TUG1 and DDX were positively correlated in CRC tissues. qRT-PCR suggested that overexpression of TUG1 or IGF2BP2 (vs. the vector group) remarkably enhanced the profiles of HDGF and DDX in LoVo and HCT15 cells. Additionally, by contrast to the si-NC group, when the profile of IGF2BP2 was knocked down, their expressions in those cells were abated (*P < 0.05*, Figure 4(g-h)). According to the above discoveries, HDGF and DDX expressions were heightened in CRC tissues and were positively relevant to TUG1’s expression.

(a-b) qRT-PCR determined HDGF and DDX expressions in 43 CRC tissues and normal tissues. (c-d) HDGF and DDX protein expressions in 3 CRC tissues were figured out by Western blot. ***P < 0.001 (vs. the Normal group). (e-f) The analysis of the correlation between TUG1 and HDGF, as well as between TUG1 and DDX in CRC tissues. g-h: qRT-PCR confirmed the profiles of HDGF and DDX in CRC cells following overexpression or knockdown of TUG1 or IGF2BP2. ** P < 0.01, ***P < 0.001 (vs. the vector group). *##P < 0.01* (vs. the si-NC group). N = 3.

### 3.5 HDGF knockdown weakened IGF2BP2/TUG1-mediated cisplatin resistance

**Figure 5.**
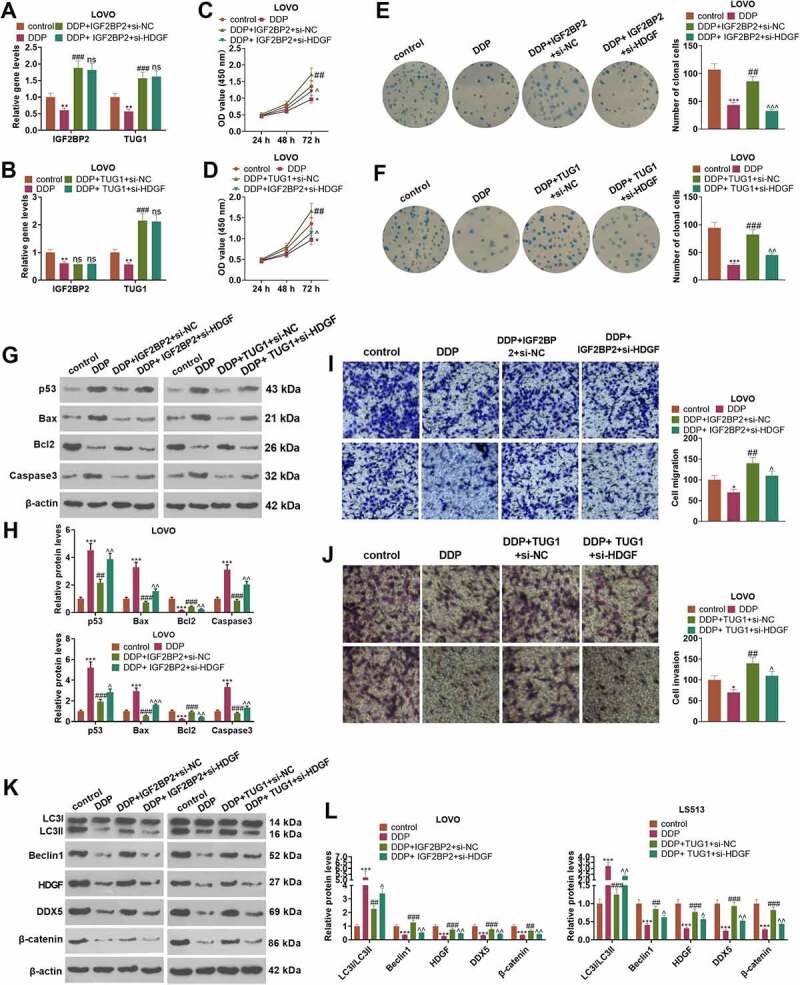
HDGF knockdown alleviated IGF2BP2/TUG1-mediated cisplatin resistance.

To figure out the mechanism of how IGF2BP2/TUG1 would influence cisplatin resistance, we transfected LoVo cells along with IGF2BP2/TUG1+ si-NC and IGF2BP2/TUG1+ si-HDGF. Then DDP was taken to treat the cells, with a blank control group (con) set up. As displayed in Figure 5(a,b), in contrast with the control group, DDP greatly hampered the profiles of IGF2BP2 and TUG1 in LoVo cells (*P < 0.05*). In contrast with the DDP group, overexpression of IGF2BP2 substantially enhanced TUG1’s expression (*P < 0.05*), while TUG1 overexpression exerted no distinct influence on the profile of IGF2BP2 (*P > 0.05*). By contrast to the DDP+IGF2BP2+ si-NC group or DDP+TUG1+ si-NC group, HDGF knockdown contributed to no evident alterations in IGF2BP2 and TUG1 in LoVo cells (*P > 0.05*). CCK8 displayed that in contrast with the DDP+IGF2BP2+ si-NC or DDP+TUG1+ si-NC group, HDGF inhibition evidently suppressed LoVo cell proliferation (*P < 0.05*, Figure 5(c-d)). Colony formation assay revealed that HDGF knockdown remarkably weakened the promoting impact of IGF2BP2/TUG1 overexpression on the colony formation ability of the cells (*P < 0.05*, Figure 5(e-f)). Western blot disclosed that when HDGF was knocked down following overexpression of TUG1, DDP’s ability to trigger LoVo cell apoptosis was notably attenuated (*P < 0.05*, Figure 5(g-h)). Transwell denoted that in contrast with the DDP+IGF2BP2/TUG1+ si-NC group, knockdown of HDGF distinctly impeded LoVo cells’ migration and invasion (*P < 0.05*, Figure 5(i-j)). As presented in Figure 5(k,l), Western blot unveiled that in contrast with the DDP+IGF2BP2/TUG1+ si-NC group, HDGF knockdown gave rise to a dramatic rise in the level of LC3I/LC3II and a decline in the protein profiles of Beclin1 and the HDGF/DDX5/β-catenin axis. Collectively, those data supported that HDGF knockdown repressed IGF2BP2 or TUG1 overexpression-mediated DDP resistance.

LoVo cells were transfected along with IGF2BP2/TuG1+ si-NC and IGF2BP2/TuG1 + si-HDGF, followed by cell treatment with DDP (4 g/ml) for 24 hours. The blank control (con) group was established. (a-b) The profiles of IGF2BP2 and TUG1 in LoVo cells were determined through qRT-PCR. (c-d) The proliferation of LoVo cells was monitored through CCK8. (e-f) Colony formation assay evaluated the colony formation capability of LoVo cells. (g-h) Western blot measured p53, Bax, Bcl2, and Caspase3 in LoVo cells. (i-j) Cell migration was tracked by Transwell. (k-l) The protein profiles of autophagy-concerned proteins (LC3I/LC3II, Beclin1) and the HDGF/DDX5/β-catenin pathway were verified through Western blot. *P < 0.05, **P < 0.01, ***P < 0.001 (vs. the con group). *nsP>0.05, #P < 0.05, ##P < 0.01, ###P < 0.001* (vs. the DDP group). *^P < 0.05, ^^P < 0.01, ^^^P < 0.001* (vs. the DDP+IGF2BP2+ si-NC group). N = 3.

### 3.6 TUG1 targeted miR-195-5p, and miR-195-5p targeted HDGF

**Figure 6.**
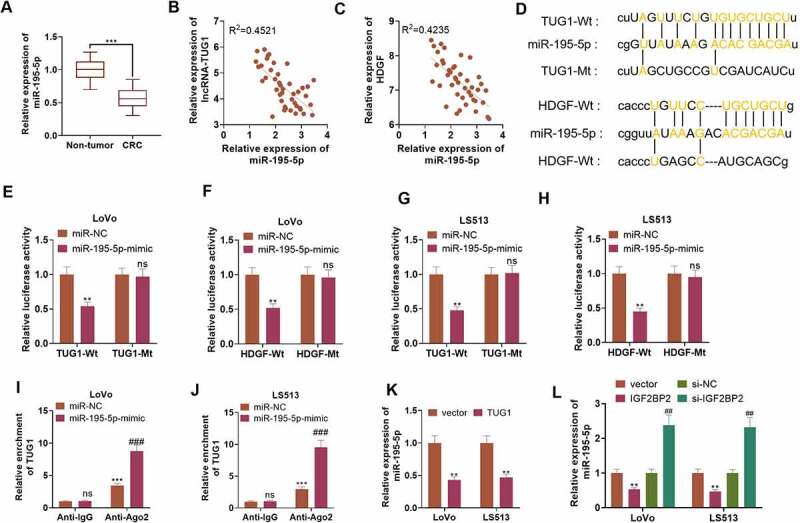
TUG1 targeted miR-195-5p and miR-195-5p targeted HDGF.

qRT-PCR ascertained the profile of miR-195-5p, indicating that the profile was notably lowered in 43 CRC tissues as opposed to adjacent non-tumor tissues (*P < 0.05*, Figure 6(a)). As per Pearson analysis, the profiles of miR-195-5p and TUG1, as well as miR-195-5p and HDGF were negatively relevant in CRC tissues (Figure 6(b-c)). The base complementary sequences of TUG1 and miR-195-5p, and miR-195-5p and HDGF were uncovered in the ENCORI database (http://starbase.sysu.edu.cn/) (Figure 6(d)). Dual-luciferase assay suggested that in contrast with the miR-NC group, overexpression of miR-195-5p considerably hindered the luciferase activity of LoVo and HCT15 cells transfected together with TUG1-Wt or HDGF-Wt (*P < 0.05*, Figure 6(e-h)). RIP displayed that by contrast to the miR-NC group, overexpression of miR-195-5p substantially stepped up TUG1 enrichment in LoVo and HCT15 cells in the anti-Ago2 group (*P < 0.05*, Figure 6(i-j)). As exhibited in Figure 6(k), qRT-PCR revealed that TUG1 overexpression vigorously brought down the profile of miR-195-5p in LoVo and HCT15 cells (*P < 0.05*). Figure 6(l) reflected that IGF2BP2 overexpression prominently repressed miR-195-5p’s expression, whereas IGF2BP2 knockdown bolstered its expression (*P < 0.05*). These findings signified that TUG1 targeted and negatively modulated miR-195-5p, which targeted and negatively regulated HDGF’s expression.

(a) qRT-PCR verified the profile of miR-195-5p in CRC tissues and non-tumor tissues. ***P < 0.001 (vs. the Non-tumor group). (b-c) Pearson analysis ascertained the correlation between miR-195-5p and TUG1, miR-195-5p and HDGF in CRC tissues. (d) The base complementary sequences of TUG1 and miR-195-5p as well as miR-195-5p and HDGF were discovered in the ENCORI database (http://starbase.sysu.edu.cn/). (e-h) Dual-luciferase assay defined the function of miR-195-5p in the wild type (Wt) and mutant type (Mt) of TUG1 and HDGF. (i-j) The binding correlation between TUG1 and HDGF was assessed via RIP assay. nsP > 0.05, **P < 0.01, ***P < 0.001 (vs. the Anti-IgG group), *###P < 0.001* (vs. the miR-NC group). (K) miR-195-5p expression following overexpression of TUG1 was disclosed by qRT-PCR. (L) The influence of IGF2BP2 overexpression or knockdown on miR-195-5p’s expression was uncovered through qRT-PCR. **P < 0.01 (vs. the vector group). *##P < 0.01* (vs. the si-NC group). N = 3.

### 3.7 miR-195-5p curbed CRC cells’ resistance to cisplatin through autophagy inhibition

**Figure 7.**
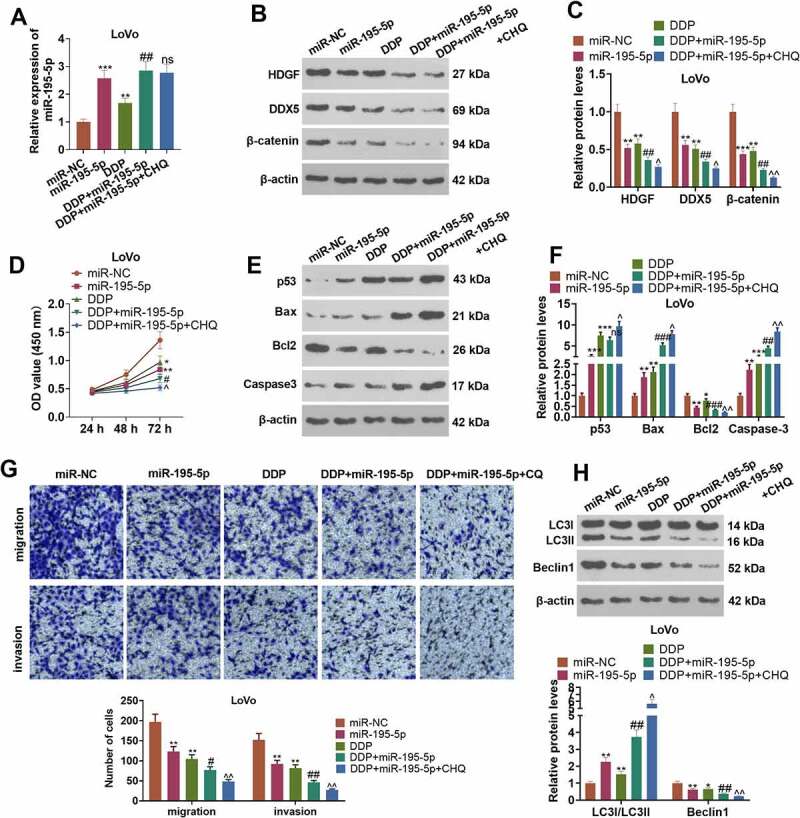
miR-195-5p impeded CRC cells’ resistance to cisplatin via autophagy inhibition.

To comprehend the mechanism of miR-195-5p influencing CRC cisplatin resistance, we transfected LoVo cells along with miR-NC and miR-195-5p mimics. CQ was taken to treat the cells following the transfection. DDP was adopted to intervene in the cells. qRT-PCR displayed that in contrast with the miR-NC or DDP group, transfected miR-195-5p mimics contributed to a rise in the profile of miR-195-5p (*P < 0.05*, Figure 7(a)). Western blot revealed that miR-195-5p mimics vigorously drove up the profiles of HDGF, DDX5, and β-catenin (vs. the miR-NC or DDP group) (*P < 0.05*, Figure 7(b-c)). CCK8 and Western blot examined cell proliferation and apoptosis. As compared with the miR-NC or DDP group, overexpression of miR-195-5p distinctly impeded LoVo cells’ proliferation and facilitated their apoptosis (*P < 0.05*, Figure 7(d-f)). Transwell displayed that in contrast with the miR-NC or DDP group, overexpression of miR-195-5p triggered a conspicuous decline in the migration and invasion of the cells (*P < 0.05*, Figure 7(g)). Nevertheless, in contrast with the DDP+miR-195-5p group, CQ barely influenced miR-195-5p’s expression and LoVo cell development. Western blot verified the profiles of autophagy-concerned proteins. When compared to the miR-NC or DDP group, overexpression of miR-195-5p lowered the level of LC3I/LC3II and raised the profile of Beclin1. By contrast to the DDP+miR-195-5p group, under the influence of CQ, the ratio of LC3I/LC3II was elevated, and Beclin1’s expression was hampered (*P < 0.05*, Figure 7(h)). These findings disclosed that miR-195-5p suppressed autophagy and weakened CRC cells’ resistance to cisplatin.

LoVo cells were transfected along with miR-NC and miR-195-5p. Then, CQ (20 µM) and DDP (4 µg/ml) were adopted to treat the cells for 24 hours. (a) qRT-PCR determined the profile of miR-195-5p. (b-c) Western blot checked the profiles of HDGF, DDX5, and β-catenin. D: CCK8 examined cell proliferation. (e-f) Western blot measured p53, Bax, Bcl2, and Caspase3 in LoVo cells. (g) Transwell tracked cell migration. h-i: Transwell monitored cell invasion. (h) The profiles of autophagy-linked proteins (LC3I/LC3II, Beclin1) were confirmed via Western blot. *P < 0.05, **P < 0.01, ***P < 0.001 (vs. the miR-NC group). *#P < 0.05, ##P < 0.01* (vs. the DDP group*). nsP>0.05, ^P < 0.05, ^^P < 0.01* (vs. the DDP+miR-195-5p group). N = 3.

### 3.8 Overexpression of miR-195-5p impaired the cancer-promoting function of TUG1

**Figure 8.**
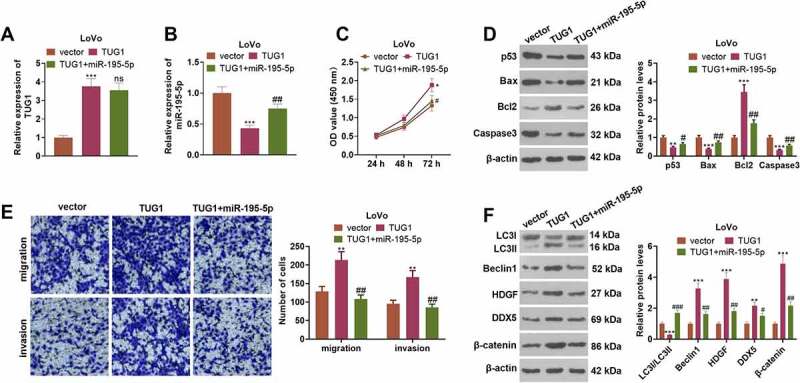
Overexpression of miR-195-5p weakened the cancer-promoting function of TUG1.

To clarify the mechanism of TUG1 boosting CRC cell growth, we transfected LoVo cells along with the plasmid TUG1, its negative control vector, and TUG1+ miR-195-5p. As indicated by qRT-PCR, in contrast with the vector group, overexpression of TUG1 greatly lowered the profile of miR-195-5p (*P < 0.05*, Figure 8(a-b)). By contrast to the TUG1 group, overexpression of miR-195-5p exerted little influence on TUG1’s expression (*P > 0.05*, Figure 8(a-b)). CCK8 denoted that in contrast with the TUG1 group, miR-195-5p mimics substantially dampened LoVo cell proliferation (*P < 0.05*, Figure 8(c)). As presented in Figure 8(d-f), overexpression of miR-195-5p resulted in an increase in the apoptosis of LoVo cells and a decrease in their migration and invasion levels in contrast with the TUG1 group (*P < 0.05*). Overexpression of miR-195-5p notably heightened the level of LC3I/LC3II and curbed the profiles of Beclin1 and the HDGF/DDX5/β-catenin pathway (*P < 0.05*, Figure 8(f)). All the discoveries unraveled that overexpression of miR-195-5p attenuated the promoting impact of TUG1 on LoVo cells.

LoVo cells were transfected along with the plasmid TUG1, its negative control vector, and TUG1+ miR-195-5p. (a-b) miR-195-5p and TUG1 expressions were determined through qRT-PCR. (c) CCK8 checked cell proliferation. d: Western blot examined p53, Bax, Bcl2, and Caspase3 in LoVo cells. (e) Transwell tracked migration and invasion. (f) The protein profiles of autophagy-associated proteins (LC3I/LC3II, Beclin1) and the HDGF/DDX5/β-catenin axis were ascertained by Western blot. *P < 0.05, **P < 0.01, ***P < 0.001 (vs. the vector group). *nsP>0.05, #P < 0.05, ##P < 0.01* (vs. the TUG1 group). N = 3.

### 3.9 Overexpressed IGF2BP2 boosts tumorigenesis in colon cancer cells

**Figure 9.**
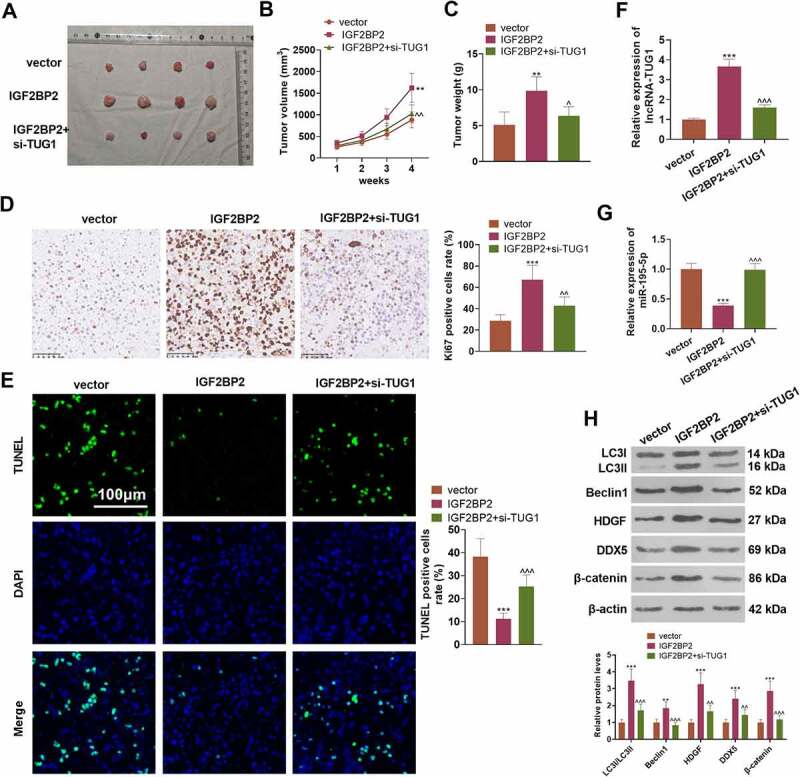
Overexpression of IGF2BP2 boosted tumor formation in colon cancer cells.

To understand the influence of IGF2BP2 and TUG1 on CRC cell growth *in vivo*, the stably transfected LoVo cell suspension was utilized to engineer a nude mouse model. Figure 9(a) presented the tumor tissues extracted out of the dead nude mice. In contrast with the vector group, overexpression of IGF2BP2 substantially enlarged the tumor volume and mass. As compared with the IGF2BP2 group, TUG1 knockdown vigorously dampened tumor growth (*P < 0.05*, Figure 9(b-c)). IHC signified that IGF2BP2 overexpression dramatically uplifted the proportion of positive Ki67 cells, while TUG1 knockdown or miR-195-5p overexpression (compared to the IGF2BP2 group) considerably suppressed that of the cells (*P > 0.05*, Figure 8(d)). TUNEL examined cell apoptosis, indicating that in contrast with the vector group, overexpression of IGF2BP2 gave rise to a sharp drop in the number of positive TUNEL cells. However, in contrast with the IGF2BP2 group, TUG1 knockdown or miR-195-5p overexpression led to a remarkable increase in the number of positive TUNEL cells (*P < 0.05*, Figure 9(e)). qRT-PCR revealed that overexpression of IGF2BP2 considerably enhanced TUG1 but restrained the profile of miR-195-5p, whereas TUG1 knockdown or overexpression evidently heightened miR-195-5p’s expression (vs. the IGF2BP2 group *P < 0.05*, Figure 9(f-g)). Western blot displayed that compared to the vector group, IGF2BP2 overexpression distinctly limited the ratio of LC3I/LC3II and elevated the protein profiles of Beclin1 and the HDGF/DDX5/β-catenin axis. In contrast with the IGF2BP2 group, following knockdown of TUG1 or overexpression of miR-195-5p, the level of LC3I/LC3II was greatly stepped up, as the profiles of Beclin1 and the HDGF/DDX5/β-catenin axis were markedly abated (*P < 0.05*, Figure 9(h)). These findings unveiled that overexpression of IGF2BP2 boosted tumor formation and triggered autophagy in the LoVo cells of nude mice dependently on TUG1.

The LoVo cell suspension, stably transfected along with the vector, IGF2BP2, IGF2BP2+ si-TUG1, and IGF2BP2+ miR-195-5p, was subcutaneously transfused into the nude mice for the construction of a nude mouse model. (a-c) The tumor histogram. The tumor volume and weight were counted. (d) IHC calculated the proportion of positive Ki67 cells. (e) TUNEL examined cell apoptosis. (f-g) qRT-PCR verified TUG1 and miR-195-5p expressions in the tumors. (h) The protein profiles of autophagy-concerned proteins (LC3I/LC3II, Beclin1), and the HDGF/DDX5/β-catenin pathway were determined through Western blot. **P < 0.01, ***P < 0.001 (vs. the vector group). *^P < 0.05, ^^P < 0.01, ^^^P < 0.001*, (vs. the IGF2BP2 group). N = 5.

## 4. Discussion

Chemotherapy resistance of tumors is one of the leading contributors to tumor growth and recurrence [35]. Autophagy, a self-degradation system, exists extensively in the cure for sensitive and drug-resistant cancers [36]. During the process of autophagy, a lysosome eliminates damaged cellular components to maintain intracellular circulation and homeostasis [37]. Autophagy, which plays a dynamic role in carcinogenesis or tumor suppression in different phases of tumors, boasts the ability to curb the occurrence and development of early-stage tumors and to boost the growth and metastasis of late-stage tumors [38]. Preceding studies have denoted that autophagy is inextricably correlated with the occurrence and progression of CRC [39]. Here, we discovered that autophagy inhibition could suppress the survival of CRC cells in an *in-vitro* culture environment.

Reportedly, IGF2BP2 serves as an oncogene in various solid cancers [40]. For instance, IGF2BP2 presents a high expression in liver cancer tissues and facilitates liver cancer development via the m6a-Flap endonuclease 1 (FEN1)-dependent mechanism [41]. IGF2BP2, overexpressed in pancreatic cancer tissues, boosts pancreatic cancer cells’ growth through the activation of the PI3K/Akt signaling pathway *in vitro* and *in vivo* [42]. IGF2BP2 can obstruct miR-195 from degrading RAF1, thus facilitating the proliferation and survival of CRC cells [43]. This signifies that IGF2BP2 exerts a cancer-promoting influence on CRC. Here, we discovered that in contrast with normal tissues or cells, the profile of IGF2BP2 was uplifted in both CRC tissues and cells. Overexpression of IGF2BP2 strengthened CRC cells’ resistance to cisplatin *in vivo* via boosting cell proliferation and autophagy as well as triggering apoptosis. This further cemented the status of IGF2BP2 as an oncogene in CRC. Additionally, the interplay between RNA and proteins could participate in the regulation of multiple important biological processes [44]. lncRNA LINRIS stabilizes IGF2BP2 to boost the aerobic glycolysis of CRC cells, hence facilitating the evolvement of the cancer [45]. That is to say, IGF2BP2 can modulate tumor development via its interaction with RNA. According to the database analysis, the profiles of IGF2BP2 and TUG1 are positively relevant in both Colon adenocarcinoma (COAD) or Rectum adenocarcinoma (READ), which means they may well interact with each other in CRC. Interestingly, our work unraveled IGF2BP2’s boost to TUG1’s expression. This denotes that the cancer-promoting function of IGF2BP2 in CRC is achieved at least partially through the regulation of TUG1’s profile.

TUG1, aberrantly expressed in CRC, takes part in the cancer’s progression. For instance, TUG1 can facilitate CRC cell proliferation, metastasis, and epithelial-mesenchymal transformation via the miR-138-5p/ZEB2 zinc finger E-box binding homeobox 2 (ZEB2) axis [46]. It can also sponge miR-145-5p to elevate the profile of transient receptor potential channel 6 (TRPC6), hence boosting the growth and metastasis of CRC cells [47]. In line with the base complementary sequence of TUG1 and miR-195-5p, our work uncovered a targeting correlation between them via dual-luciferase assay and RIP. Studies have corroborated that miR-195-5p, down-regulated in CRC tissues and related to poor prognosis in CRC sufferers, modulates the notch receptor 2 (NOTCH2)-mediated epithelial-mesenchymal transformation to influence M2-like macrophage polarization in the cancer [48]. Through qRT-PCR, it was revealed that the profile of miR-195-5p was remarkably lower in CRC cancer tissues than in paracancerous normal tissues, which is aligned with the above studies. The compensation assay was implemented, indicating that overexpression of miR-195-5p weakened the cancer-promoting function mediated by TUG1, which means TUG1 serves as a sponge of miR-195-5p to facilitate the *in-vitro* growth and cisplatin resistance of CRC cells.

HDGF, DDX, and β-catenin present aberrant expressions in a multitude of tumors. For instance, HDGF down-regulation limits the profile of the PI3K-Akt signaling pathway, dampening bladder cancer cell growth *in vitro* and in xenograft nude mouse models [49]. That means HDGF functions as an oncogenic gene in the context of bladder cancer. Similarly, we discovered that HDGF, down-regulated in CRC tissues, was a downstream target of miR-195-5p. Additionally, miR-182 triggers the Wnt/β-catenin signaling pathway to boost the proliferation, migration, and invasion of prostate cancer cells and curb their apoptosis [50]. NEAT1 initiates Wnt/β-catenin signal transduction via the stabilization of protein DDX5, thus stepping up CRC cells’ proliferation and metastasis [51]. Our work also disclosed that the profile of DDX5 was heightened in CRC tissues and that overexpression of GF2BP2 or TUG1 bolstered its expression in CRC cells. Importantly, we uncovered that HDGF down-regulation could abate IGF2BP2/TUG1-mediated CRC cisplatin resistance through autophagy inhibition and suppress the profiles of DDX5 and β-catenin. Nonetheless, more studies are still in need to further investigate other molecular mechanisms of TUG1 affecting colorectal cancer.

## 5. Conclusion

**Figure 10.**
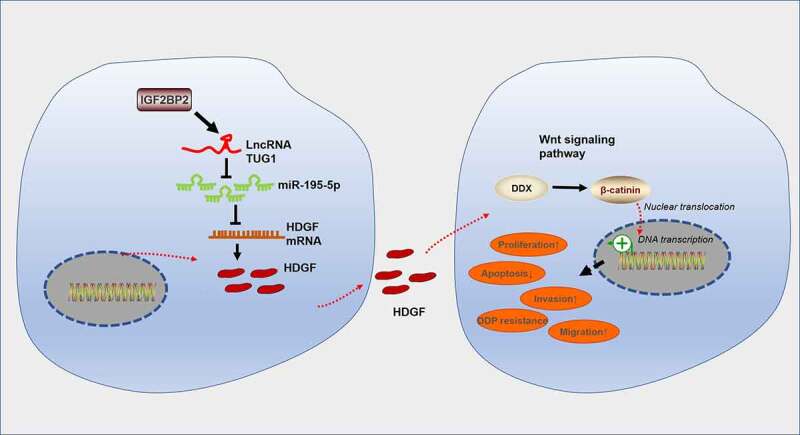
The mechanism diagram of the IGF2BP2/TUG1/miR-195-5p/HDGF axis in colorectal cancer progression and chemoresistance.

To summarize, our study has figured out the oncogenic function of IGF2BP2-stabilized TUG1 in colorectal cancer. A deeper mechanical exploration has pinpointed that TUG1 targets miR-195-5p and hampers its expression. miR-195-5p boosts the HDGF/DDX/β-catenin axis to trigger autophagy, thus stepping up CRC cells’ growth *in vitro* and *in vivo* as well as their resistance to cisplatin (Figure 10). This hints that the IGF2BP2/TUG1 axis is an underlying target for colorectal cancer treatment.

## Data Availability

The data sets used and analyzed during the current study are available from the corresponding author on reasonable request (https://www.haodf.com/doctor/6132737783.html).

## Abbreviations

DDP: Cisplatin; CRC: colorectal cancer; lncRNAs: long non-coding RNAs; TUG1: Taurine upregulated gene 1; IGF2BP2: Insulin-like growth factor 2 mRNA-binding protein 2; CQ: Chloroquine; CCK8: Cell Counting Kit-8; RIP: RNA immunoprecipitation; MALAT1: lncRNA metastasis-associated lung adenocarcinoma transcript 1; miRNAs: microRNAs; LncRNA MALAT1:lncRNA metastasis-associated lung adenocarcinoma transcript 1; lncRNA DLEU2: lncRNA Deleted in Lymphocytic Leukemia 2; lncRNA BANCR: BRAF-activated non-protein coding RNA; CARM1: Coactivator-associated arginine methyltransferase 1; HDGF: Hepatoma-derived growth factor; DDX5: DEAD-box RNA helicase DDX5; RBPs: RNA-binding proteins; qRT-PCR: Quantitative real-time polymerase chain reaction; OD: opticaldensity; CO_2_: carbon dioxide; PVDF: polyvinylidene difluoride; SDS-PAGE: sodium dodecyl sulfate-polyacrylamide gel electrophoresis; TBST: TBS with Tween-20; WT: wild type. MT: Mutant; IHC: Immunohistochemistry; PBS: phosphate-buffered saline; TUNEL: TdT-mediated dUTP Nick-End Labeling; FEN1: Flap endonuclease 1; COAD: Colon adenocarcinoma; READ: Rectum adenocarcinoma; ZEB2: zinc finger E-box binding homeobox 2; TRPC6: transient receptor potential channel 6; NOTCH2: notch receptor 2;
